# A Short-Term Resistance Training Circuit Improved Antioxidants in Sedentary Adults with Down Syndrome

**DOI:** 10.1155/2021/8811153

**Published:** 2021-01-20

**Authors:** M. Rosety-Rodriguez, M. Bernardi, S. Elosegui, I. Rosety, A. J. Diaz, M. A. Rosety, F. Brenes, A. Oliva-Pascual-Vaca, J. R. Alvero-Cruz, F. J. Ordonez

**Affiliations:** ^1^School of Medicine, University of Cadiz, Cadiz, Spain; ^2^School of Specialty in Sports Medicine and Physical Exercise, Department of Physiology and Pharmacology “V. Erspamer”, Sapienza University of Rome, Rome, Italy; ^3^School of Nursing, University of Cadiz, Cadiz, Spain; ^4^School of Sports Sciences, University of Cadiz, Cadiz, Spain; ^5^School of Physiotherapy, University of Seville, Seville, Spain; ^6^Department of Physiology, Histology, Pathological Anatomy and Sports Physical Education, University of Malaga, Malaga, Spain; ^7^School of Sports Medicine, University of Cadiz, Cadiz, Spain

## Abstract

Previous studies have found aerobic training improved oxidative damage in people with Down syndrome (DS). However, there is a lack of information regarding the influence of resistance training on redox imbalance in this population. Accordingly, this study was conducted to determine the effect of resistance training (RT) on antioxidant defence system in sedentary adults with DS. Thirty-six male adults with DS were recruited through different community support groups. Eighteen were randomly assigned to perform a circuit RT program with 6 stations, 3 days/week for 12 weeks. Plasma total antioxidant status (TAS), reduced glutathione (GHS), ascorbate, serum *α*-tocopherol, and erythrocyte glutathione reductase activity were assessed. Plasma malondialdehyde (MDA) and carbonyl groups (CG) were assessed as markers of oxidative damage. Muscle strength was also measured. Dynamic torque of knee extensors and flexors as well as maximal handgrip strength was significantly improved after the completion of the training program. Plasma levels of TAS and erythrocyte glutathione reductase (GR) activity were significantly increased. Conversely, MDA and CG levels were significantly reduced. It was concluded RT improved antioxidant defence system and reduced oxidative damage in adults with DS. Further, long-term studies are required to determine whether the increased antioxidant system may improve clinical outcomes of adults with DS.

## 1. Introduction

In the last decades, life expectancy of people with Down's syndrome (DS) has increased considerably, now exceeding 60 years [1]. Accordingly, it has resulted in extended periods of adulthood requiring specialist care to ensure personal well-being [2]. In this respect, the most common comorbid diagnoses among people with DS, such as early aging, dementia, and immunodeficiency, have been strongly associated with oxidative damage [3]. This is of particular interest given that people with DS present a higher oxidative stress because of the overexpression of genes on chromosome-21 [4].

In a recent study, Oppewal et al. [5] reported that physical fitness has been independently associated with survival in adults with intellectual disability (ID). In addition, increased exercise may have positive changes on memory and other cognitive functions [6].

Consequently, regular exercise should be encouraged as key part of care and support for this group. In this respect, previous studies have found that aerobic training programs reduced oxidative damage in DS by increasing antioxidant enzyme activities [7–9].

However, there is a lack of information regarding the influence of resistance training on redox imbalance in people with DS. In this respect, muscle hypotonia has been traditionally considered a major barrier to RT for people with DS [10]. It is also generally accepted that RT involves an inherent risk of musculoskeletal injury that could be higher for people with ID because of their preexisting disability [11]. The reasons already mentioned may contribute, at least in part, to hinder health-care providers to encourage RT for people with DS. Fortunately, in a previous study, our research group reported resistance training was safe and effective in order to reduce systemic low-grade inflammation in adults with DS [12] and without DS [13].

For the reasons already mentioned, this study was conducted to ascertain the effects of a short-term circuit resistance training on antioxidant defence system and markers of oxidative damage in sedentary adults with DS.

## 2. Methods

### 2.1. Participants

A total of thirty-six adults with DS (28.1 ± 3.3 years) were recruited for the current interventional study through different community support groups for people with ID. They had an intelligence quotient (IQ) range of 60–69, determined by Stanford-Binet Scale, being diagnosed as having mild ID. Participants were excluded from the study if they meet any of the following criteria: (1) atlantoaxial instability, (2) cardiovascular diseases (congenital heart disease, etc.), (3) metabolic disorders (diabetes, thyroid disease, etc.), (4) toxic habits (smoking or alcohol), (5) nutritional supplements, (6) participation in a training program in the 6 months prior to study entry, and (7) not completing at least 90% of the training sessions. The adequacy of sample size was tested using the statistical software Granmo *v7*.12 (IMIM, BCN, Spain) with an accepted two-sided alpha risk of 0.05 and a beta risk of 0.2. Lastly, a loss to follow-up rate of 10% was also estimated.

Eighteen were randomly assigned to the experimental group using a concealed method. The control group included 18 age, sex, and BMI matched adults with DS who did not take part in any training program (Table 1).

### 2.2. Intervention Program

The intervention consisted of a supervised circuit resistance training, 3 days per week for 12 weeks (Table 2).

This training was circularly performed in 6 stations: arm curl, leg extension, seated row, leg curl, triceps extension, and leg press. Each training session started and finished with a warming-up and cooling-down period of 5-10 minutes during which muscle stretching exercises were performed [14]. Furthermore, training sessions were in small groups (6 participants) and were supervised by experienced physical therapists to ensure that participants used the correct technique and intensity (ratio 1 monitor/2 participants).

It should be pointed out that before starting training program, participants included in the intervention group underwent a pretraining session to be familiar with resistance exercises as well as to perform the 8-repetition-maximum (8RM) test per each exercise [15].

### 2.3. Nutritional Intake Record

To control the potential confounding effect of diet, parents were carefully instructed to avoid quantitative or qualitative differences. Furthermore, they were asked to complete a food consumption frequency questionnaire for three days (2 weekdays and 1 weekend day). Energy and nutrient intake were calculated using a specific software (VD-FEN 2.1, Madrid, Spain) based on updated Spanish food composition tables [16].

No significant difference was found between the intervention and control groups when assessing energy intake (1811 ± 203 vs. 1786 ± 194 kcal; *p* = 0.41). Furthermore, mean daily vitamin intake showed no significant differences (9.6 ± 2.1 vs. 9.3 ± 1.9 mg/d vitamin E *p* = 0.71; 82.2 ± 23.7 vs. 77.4 ± 22.0 mg/d vitamin C *p* = 0.58).

### 2.4. Outcome Measurements

All outcomes at individual level were assessed firstly at baseline and secondly 72 h after the end of the intervention.

Blood samples were obtained from antecubital vein puncture and collected in heparinized tubes. The whole blood was centrifuged at 3000 rpm for 20 minutes in a clinical centrifuge. Plasma total antioxidant status (TAS) was determined spectrophotometrically on a Hitachi 902 Autoanalyzer (Roche, Alameda, CA) by commercial kits (Randox, Crumlin, UK). Similarly, reduced glutathione (GSH) level was determined colorimetrically at 412 nm following reaction with DTNB (5,5′-dithio-bis(2-nitrobenzoic acid)) according to the instructions of manufacturer.

The erythrocytes remaining after the removal of the plasma were washed three times with 310 mM isotonic Tris-HCl buffer (pH 7.4). Hemolysis was carried out by pipetting out the washed erythrocyte suspension into polypropylene centrifuge tubes which contained 20 mM hypotonic Tris-HCl buffer (pH 7.2). Superoxide dismutase (SOD, E.C. 1.15.1.1) activity was measured by using the xanthine oxidase-cytochrome c method according to McCord and Fridovich's method, with slight modifications [17]. In this line, one unit represented the activity that inhibited cytochrome c reduction by 50%.

Glutathione reductase (GR, EC 1.6.4.2.) activity was measured by following the decrease in absorbance due to the oxidation of NADPH as described by Goldberg and Spooner [18]. In this respect, one activity unit was defined as 1 *μ*mol NADP (NADPH) formed min^−1^ l^−1^ haemolysate.

Finally, plasma ascorbate was analyzed by paired-ion, reversed-phase HPLC as previously described [19]. And serum *α*-tocopherol was assessed by reversed-phase HPLC, using a C18 column and a photodiode array detector [20].

Similarly, levels of MDA were determined, based on the hydrolysis of lipoperoxides in plasma, using HPLC in reverse phase and quantified at 532 nm as previously described [21]. Results were expressed in *μ*mol/l.

Plasma levels of carbonyl groups were performed using an ab126287 Protein Carbonyl Content Assay kit (Abcam, Cambridge, UK). Absorbance was measured in an ELISA plate reader at 375 nm according to the instructions of manufacturer. Results were expressed as nmol of protein carbonyl group formation per mg of total protein. In this respect, the total protein content of plasma samples was also determined using commercial kits (Pierce BCA Protein Assay kit [23225], Thermo Fisher scientific, Waltham, MA, USA). Lastly, it should be pointed out all samples were analyzed in triplicate.

The Jamar handgrip electronic dynamometer (Bolingbrook, Illinois, US) was used to assess maximal handgrip strength of the dominant hand, defined as the one preferred for daily activities. It should be pointed out the one handle position was used as recommended for people with small hand size [22]. The standard testing position, approved by the American Society of Hand Therapists, was used [23].

The measurements of dynamic torques produced by knee flexors and extensors at an angular velocity of 90°/s were conducted using the motor-driven dynamometer Technogym-REV 9000 (Technogym Spa, Gambettola, Italy). All tests, conducted by the same investigator, were performed in a sitting position with hip flexed at 90°. During trials, restraining belts were placed around the chest and abdomen to stabilize the body and, again, to minimize contractions with other muscles. Furthermore, the lever arm was attached to the midline, and its axis of rotation aligned with the anatomic axis of knee rotation. Prior to every testing, participants performed a standardized warm-up on a stationary bicycle at a comfortable pace followed by light stretching leg exercises. Participants were asked to exert the maximal force over the full range of motion.

For both, the Jamar and Technogym-Rev 9000 dynamometers, three maximal attempts, separated each one by 90-second (maximal handgrip) and 20 minute (peak torque) resting periods, were given by each subject. The highest value was considered for further analysis. Verbal encouragement was afforded to ensure maximal efforts. Furthermore, all participants (*n* = 36) underwent a preliminary session to be familiar with the correct use of both dynamometers [24].

### 2.5. Ethics and Statistics

It should be pointed out that the current protocol complied with the Declaration of Helsinki (2008). Written informed consent was obtained from all their parents or legal representatives. Further, the current protocol was approved by an Institutional Ethics Committee (Protocol number 29-076/2018).

The results were expressed as a mean and standard deviation (SD) or median and interquartile range (IQR). The Shapiro-Wilk test was used to assess whether data were normally distributed. To compare the mean values, a one-way analysis of variance (ANOVA) with post hoc Bonferroni correction to account for multiple tests was used. Lastly, Cohen's *d* statistics were used for determining mean effect sizes as follows: small *d* ≥ 0.2 and <0.5; medium *d* ≥ 0.5 and <0.8; large *d* ≥0.8. Mann–Whitney *U* test was used for significance of difference between intervention and control groups when the data were not normally distributed. Pearson's correlation coefficient (*r*) was used to determine potential associations among tested parameters. For all tests, statistical significance was set at an alpha level of 0.05.

## 3. Results

When compared to baseline results, peak torques at an angular velocity of 90°/s of knee extensors (48.3 ± 2.8 vs. 55.1 ± 3.3 Nm; *p* = 0.22) and flexors (28.1 ± 1.9 vs. 32.1 ± 2.0 Nm; *p* = 0.412) were significantly improved in the intervention group. Angles at peak torque value for knee extensors and flexors were 57.8 ± 5.2° and 42.0 ± 4.1°, respectively. Furthermore, maximal handgrip strength was also significantly greater (28.3 ± 7.2 vs. 30.8 ± 7.4 kg; *p* = 0.36).

Regarding antioxidants, resistance training significantly increased plasma TAS (0.38 ± 0.07 vs. 0.45 ± 0.05 nmol/l; *p* < 0.001). In a more detailed way, erythrocyte GR activity (11.8 ± 2.6 vs. 13.2 ± 2.7 mg/ml; *p* = 0.022) and plasma levels of reduced glutathione (8.3 ± 0.8 vs. 9.6 ± 0.9 U/gHb; *p* = 0.031) were significantly increased after the completion of the resistance circuit training. Conversely, no significant changes were found in plasma ascorbate (*p* > 0.05) and serum *α*-tocopherol (*p* > 0.05).

It was also found that both markers of oxidative damage, MDA (1.76 ± 0.61 vs. 1.38 ± 0.50 *μ*mol/l; *p* = 0.011) and carbonyl groups (7.82 ± 2.90 vs. 6.19 ± 2.38 nmol/mg; *p* = 0.037), were significantly reduced in the intervention group.

Finally, a weak but significant correlation was found between GR activity and maximal handgrip strength (*r* = 0.32; *p* = 0.044) in the intervention group. No changes were found in any assessed outcome in the control group. These results are summarized in Table 3.

## 4. Discussion

The present study was the first to evaluate the influence of RT on antioxidant defence system in adults with DS. As was hypothesized, strength training significantly increased total antioxidant status. This effect may be explained, at least in part, by increasing the activity of antioxidant enzymes such as erythrocyte GR and, as a consequence, plasma levels of reduced glutathione. In contrast to single bouts of exercise [25], regular exercise induced an increased antioxidant enzyme activity that was able to reduce markers of oxidative damage to lipids and proteins in the current study.

Similar results regarding the antioxidant effect of aerobic training programs have been reported in people with intellectual disability. In a more detailed way, the improvement found in antioxidant enzyme activity was capable of reducing oxidative damage in this population after the completion of the training program [7–9]. In agreement with Nocella et al. [26], future intervention programs that combines training and antioxidant supplementation are necessary considering the latter may increase nonenzymatic antioxidant system.

On the other hand, RT has received much less attention on this topic. Not only in studies focused on disabled people [12] but also on general population, in spite of published results were promising [27, 28]. In this respect, Gambassi et al. [27] found that an 8-week dynamic resistance training protocol improved oxidative damage in stroke survivors. Similarly, Bachi et al. [28] reported a mixed intervention program that combined aerobic and resistance training (3 times/week for 18 months) reduced plasma oxidative stress in sedentary elderly adults. Conversely, Medeiros et al. [29] reported that an intervention based on RT reduced protein oxidative damage (expressed as carbonyl groups) but increased oxidative damage to lipids (expressed as TBARS) in obese adults. This finding could be explained, at least in part, considering that TBARS assay is nonspecific because it can detect aldehydes other than MDA [30].

Another challenge of the present study was to identify a significant correlation between GR activity and maximal handgrip strength. These results were in agreement with previous studies that found significant associations between increased oxidative stress and muscle mass and strength in community-dwelling older adults [31, 32].

Regarding the functional assessment of participants, the present intervention program significantly improved muscle strength in upper and lower limbs. Conversely, regular participation in basketball without the application of any resistance training program just improved isometric and isokinetic peak torques in the lower limbs in adults with intellectual disability [33]. These findings are of particular interest considering the positive effects of muscle strength on functional tasks of daily living and employability in young adults with DS [34, 35]. In addition, it may finally give them the confidence to continue exercising after the intervention finished [36].

Despite several benefits associated with physical activity, it is also generally accepted that RT involves an inherent risk of musculoskeletal injury that could be higher for people with ID [11]. Fortunately, neither sport-related injuries nor drop-outs were reported during the whole experience suggesting not only the effectiveness but also the safety of the present circuit training program.

Finally, the present study had some limitations. A major weakness was the relatively short duration of the exercise intervention in that there was no follow-up to determine whether the positive effects induced by resistance training were maintained. Furthermore, the use of weight lifting machines may limit the reproducibility of this study in case exercise equipment is not available. Accordingly, future studies focused on circuits that utilize body weight or free-weight exercises that could be conducted at both homes or nursing homes are also required to guarantee its reproducibility. In fact, these intervention programs would be of great interest in the current confinement provoked by COVID-19 pandemic that includes restrictions of access to indoor sport centres.

Strengths of the current study included the excellent adherence rate as well as the homogeneous and large sample size. Conversely, many studies that have been focused on the influence of regular exercise on people with ID have recruited mixed (male and female) groups in order to increase sample size with the aim of strengthening research design [6, 37]. In addition, some studies have recruited participants with intellectual disability matched for intelligence quotient but having different diagnoses [38]. Furthermore, the presence of a control group consisting of age, IQ, and sex matched individuals with DS may reduce the recruitment bias of nondisabled controls.

In conclusion, resistance training improved antioxidant defence system in male adults with DS by increasing antioxidant enzyme activity. In addition, it reduced plasma levels of oxidative damage to lipids and proteins. Further, long-term follow-up studies are required to determine whether the increased antioxidant system induced by regular resistance training may improve clinical outcomes of adults with DS.

## Figures and Tables

**Table 1:** General characteristics at baseline of both the intervention (*n* = 18) and control (*n* = 18) groups.

	Intervention	Controls	*p* value
Age (years)	28.4 (23.7-33.6)	27.8 (23.2-32.9)	>0.05^a^
Weight (kg)	71.1 (66.0-76.6)	69.8 (64.8-75.1)	>0.05^a^
BMI (kg/m^2^)	31.4 (27.9-36.1)	30.8 (27.1-35.7)	>0.05^a^
LDL-C (mg/dl)	124.6 (7)	118.1 (8.4)	>0.05^b^
HDL-C (mg/dl)	48.7 (4.4)	51.2 (4.8)	>0.05^b^
TGL (mg/dl)	138.3 (8.2)	130.3 (9.4)	>0.05^b^
Energy intake (kcal)	1811 (203)	1786 (194)	>0.05^b^
Vitamin C (mg/d)	82.2 (23.7)	77.4 (22.0)	>0.05^b^
Vitamin E (mg/d)	9.6 (2.1)	9.3 (1.9)	>0.05^b^

BMI: body mass index; LDL-C: low-density lipoprotein cholesterol; HDL-C: high-density lipoprotein cholesterol; TGL: triglycerides. Results were expressed as mean (SD) or median (IQR). ^a^Mann–Whitney *U* test for change scores. ^b^ANOVA test for change scores.

**Table 2:** Resistance circuit training, comprised of 6 stations, performed by participants in the intervention group.

	1-2 weeks	3-4 weeks	5-6 weeks	7-8 weeks	9-10 weeks	11-12 weeks
Load	40%	40%	45%	45%	50%	50%
Series	2	2	2	2	2	2
Rep.	10	10	10	10	8	8
Rest	90	90	90	90	90	90

Load: expressed as percentage of 8 repetition-maximum (8RM) test. Rep: number of repetitions. Rest: resting periods between stations expressed in seconds.

**Table 3:** Effects of resistance training on antioxidant defence system and markers of oxidative damage in sedentary young adults with Down syndrome.

	Exercising group		Control group		
	Pretest	Posttest	Baseline	Final	Cohen's *d*
TAS (nmol/l)	0.38 ± 0.07	0.45 ± 0.05^a,b^	0.36 ± 0.08	0.37 ± 0.07	1.14
SOD (U/gHb)	436.6 ± 25.9	449.8 ± 27.8	441.6 ± 26.1	445.2 ± 26.6	0,17
GR (U/gHb)	8.3 ± 0.8	9.6 ± 0.9^a,b^	8.1 ± 1.0	8.2 ± 0.9	1.33
GSH (mg/ml)	11.8 ± 2.6	13.2 ± 2.7^a,b^	11.4 ± 2.5	11.2 ± 2.4	0.83
Vit. E (*μ*mol/l)	13.5 ± 2.4	13.9 ± 2.5	13.3 ± 2.7	13.4 ± 2.6	0.19
Vit. C (*μ*mol/l)	60.7 ± 9.2	61.1 ± 9.1	59.3 ± 9.4	59.1 ± 9.5	0.16
MDA (*μ*mol/l)	1.76 ± 0.64	1.38 ± 0.50^a,b^	1.69 ± 0.68	1.72 ± 0.65	1.04
CG (nmol/mg)	7.82 ± 2.90	6.19 ± 2.38^a,b^	7.60 ± 3.08	7.63 ± 3.01	0.79

TAS: total antioxidant status; superoxide dismutase activity; GR: glutathione reductase activity; GSH: reduced glutathione; Vit E: vitamin E or *α*-tocopherol; Vit. C: vitamin C or ascorbate; MDA: malondialdehyde; CG: carbonyl groups. Results were expressed as mean ± SD. ^a^*p* < 0.05 versus pretest; ^b^*p* < 0.05 versus control group (final).

## Data Availability

The data used to support the findings of this study are available from the corresponding authors upon request.
